# Application of Shark Teeth–Derived Bioapatites as a Bone Substitute in Veterinary Orthopedics. Preliminary Clinical Trial in Dogs and Cats

**DOI:** 10.3389/fvets.2020.574017

**Published:** 2020-10-28

**Authors:** Mario García-González, Fernando María Muñoz Guzón, Antonio González-Cantalapiedra, Pío Manuel González-Fernández, Rafael Otero Pérez, Julia Asunción Serra Rodríguez

**Affiliations:** ^1^Clinical Sciences Department, Veterinary Faculty, University of Santiago de Compostela, Lugo, Spain; ^2^New Materials Group, Department of Applied Physics, University of Vigo, Galicia Sur Health Research Institute (IISGS), Vigo, Spain; ^3^Traumatology and Orthopedic Surgery Unit, POVISA Hospital, Vigo, Spain

**Keywords:** hydroxyapatite, β-tricalcium phosphate, biomaterials, bone regeneration, bone substitutes, veterinary orthopedics, marine bioapatites

## Abstract

**Background:** The autograft is still considered the gold standard for the treatment of bone defects. However, given the significant morbidity of the donor site with which it has been associated, alternative substitutes for bone grafts have been developed. In the present study, a bone substitute composed of CaP biphasic bioceramics obtained from shark teeth was used (BIOFAST-VET).

**Objective:** The objective of this study is to evaluate the efficacy of a marine bioapatite in the veterinary clinical field using it as a bone-grafting scaffold in dogs and cats.

**Methods:** The biomaterial was randomly distributed in 6 veterinary clinical centers in Spain and was used in 24 cases (20 dogs and 4 cats) including 14 fractures, 9 arthrodesis, and 1 bone cyst. Grains between 500 and 2,000 μm were used. Inclusion and exclusion criteria were established. The time of consolidation and functional recovery were quantitatively and qualitatively assessed. For this, a follow-up was carried out at 2, 4, 8, and 12 weeks, included radiographic images, physical examination and sharing the feedback with the owners.

**Results:** Nineteen cases completed the study (18 dogs and 1 cat; 11 fractures, 7 arthrodesis, and 1 bone cyst). The remaining five were excluded because they did not complete the radiographic follow-up (three cats and two dogs), being three arthrodesis and two fractures. In 18 of 19 cases, the use of the biomaterial was successful; the remaining one failed due to causes not related to the biomaterial. There were no systemic or local adverse reactions. Eighteen patients had a good functional recovery. The average consolidation time was 5.94 weeks in dogs with fractures and arthrodesis, not finding statistically significant differences between sex, weight, and procedure.

**Conclusions:** This biomaterial is presented as a very suitable candidate for orthopedic surgery in the veterinary field. Preliminary results showed that its use reduces consolidation time in dogs with fractures and arthrodesis. In addition, no adverse systemic or local reactions have been observed derived from its use.

## Introduction

Bone grafts are currently very required in orthopedic medicine and dental and maxillofacial surgery for regenerating, repairing, or replacing dental pieces or bone defects. Among the main applications of bone fillings are the reconstruction of missing bone cavities, congenital malformations, or bone atrophies. Moreover, they are used to promote bone regeneration in traumatic tissue damage or injuries (1). In maxillofacial and dental surgery, bone graft scaffolds are commonly used to contribute to the suitable environment for periodontal regeneration and maxillary sinus elevation, to repair defects after teeth extraction and/or in cases of implant placement (2–4).

In the veterinary orthopedics field, the gold standard is still the utilization of fresh cancellous bone grafts for enhancing defect healing. However, over the past two decades, the application of artificial bone grafts has been augmenting (5).

Allogeneic (cadaveric) grafts and deproteinized (xenogenic) bovine bone grafts are also used because of their greater osteoconduction, along with the mechanical performance of repairing tissues (1).

In the case of allografts, they have been mostly used as substitutes for autogenous bone grafts, but low bone fusion rate and risk of disease transmission have been observed as significant problems (6). Deproteinized bovine bone has shown excellent properties because of its excellent osteoconductive capacity and biocompatibility (7), as well as a long-term remain within the matrix of the host bone (8).

These evidences justify continued sacrifices to develop effective synthetic substitutes for bone grafting. The discovery of calcium phosphate ceramics and other related biomaterials has provided a better control of the resorption process improving the capacity of the materials in the bone regeneration (4).

Hydroxyapatite (HA) is the principal inorganic constituent of vertebrate bone, and it is also lodged in the dentin and tooth enamel (9). The characteristics of HA have been investigated for several decades. Posner et al. (10) proposed its crystalline structure from the analysis of a monocrystal (11). Its ionic character makes it a stiff, refractory (12), and insoluble ceramic (13), with a melting point higher than 1,500°C (12).

This calcium phosphate, which can be acquire synthetically, has properties of biocompatibility, non-toxicity, chemical stability, osteoconduction, and bioactivity. Such properties make the material very practical for medical uses. HA can be used as a replacement for small parts of bone, reinforcement in composite materials, cavity filling in dentistry, and coating of metal surfaces for implants (14, 15).

Synthetic sources, as opposed to natural ones, in addition to the good results regarding osseointegration, provide high availability, innate reproducibility, and versatility to be integrated in concrete formulations for specific applications. Thereby, Landi et al. (16) revealed the HA has a high potential as a bone substitute replaced with strontium and magnesium in the cellular reply (16). Composite materials were proposed as another strategy to increase osteoconductivity and biocompatibility, providing an adequate resorption, like Shih et al. (17), who have tested bone generation in animal models with HA and resorbable dehydrated calcium sulfate (17). Chazono et al. (6) got a high apposition rate of minerals in rabbit bone defects of highly pure b-tricalcium phosphate (β-TCP) powder mixed with hyaluronic acid, compared to pure β-TCP in blocks (6). Bioactive glasses are very active materials that induce positive regulation of genes related to differentiation and osteoblast proliferation. As a result, they have excellent biological behavior, thanks to their osteoconductive characteristics (18, 19).

Some case reports provided details about the use of different bone substitutes in the veterinary field. One of this is a successful correction of a bone deformity where the distal tibia is turned inward toward the body (pes varus) in two Teckels. For this, a synthetic β-TCP wedge was used to fill in defects made with osteotomies. Eight weeks after the intervention, the bone was integrated. The TCP blocks were entirely resorbed after 4 months, and remodeling at the osteotomy site was observed (20, 21).

In another investigation, another method was used mixing β-TCP granules with the patient's own blood and was used as a bone substitute in defects located in long bones of 13 patients (22). The treatment of an atrophic non-union in the distal radius area of a Yorkshire Terrier was also investigated, using a three-dimensionally printed β-TCP scaffold with morphogenetic proteins (22).

Nowadays, numerous investigations are being carried out to obtain biomaterials of marine origin. The fibrillar collagen of the sea urchin has been shown to be a very valuable biomaterial for the production of skin-like scaffolds (23). Recent researches have demonstrated the possibility of obtaining bone substitutes of marine origin, BIOFAST-VET (BV). This novel product was designed to repair and regenerate bone tissue. It is made from a ceramic material obtained from the reassessment of a fish by-product, shark teeth (*Prionace glauca*). It is a very abundant and low-cost raw material at present (1, 24).

The composition, morphology, and characterization (Raman and XRD techniques) of shark teeth–derived bioapatites have been studied (1, 24–28) and revealed a globular porous structure with biphasic composing ~70% apatitic (HA, apatite-CaP, fluorapatite) and ~30% non-apatitic phase (whitlockite, β-TCP), and contributions of F, Na, and Mg. This composition and structure promoted a significantly higher bone mineral density in a rodent model after 3 weeks of healing compared with a commercial artificial biphasic HA/β-TCP (60%/40%) bone graft (*P* < 0.05). In addition, 1-mm marine bioceramics developed higher osteointegration and horizontal growth of bone tissue at the central area of the defect (1, 28).

A new advantage is that this biomaterial is environmentally friendly, because it is a natural product, gives value to fish waste, and reduces the risk of disease transmission, as it comes from a species phylogenetically far from our domestic species. Viewing the promising results (histological and radiographic) obtained in an *in vivo* study carried out on rodents (1), the mean goal of this research is to evaluate the capacity of this compound as a bone substitute in the veterinary clinical field, being used in several orthopedic procedures in dogs and cats, trying to avoid the complications derived from the non-union.

## Methods

### Biomaterial Obtaining Method

The fabrication method of the bone substitute of marine origin BV is based on pyrolytic techniques in order to remove the organic compounds. The natural precursor (shark teeth *P. glauca*) is heated to 950°C for 12 h using a heating ramp of 2°C min^−1^ and a cooling ramp of 20°C min^−1^, as described elsewhere (1, 24). Once pyrolyzed, the powders were subjected to a sieving process to select macrogranules in the diameter ranges of 0.5 to 1.0 mm, 1.0 to 2.0 mm, and 2.0 to 3.0 mm. The sterilization method used was gamma radiation (Aragogamma S.L.) (1, 24).

### Study Design

The study was designed by a multidisciplinary group made up of researchers from the School of Industrial Engineering of Vigo (PM and JA) and the Faculty of Veterinary Medicine of Lugo (A, FM, and M), with the collaboration of specialists in human orthopedic surgery (R). The material had previously passed biocompatibility tests and a complete histological evaluation in preclinical studies (1, 24, 28). The initial approach was to elucidate in which clinical situations the material object of study would be useful. Once a consensus was reached, the data collection method was designed. Afterward, a veterinary centers, clinics, and hospitals list in Spain was compiled, with sufficient orthopedic casuistry that could be included in the study. Randomly, six were selected (CV Miralbueno, Hospital Veterinario Vetpets, Hospital Veterinario Lepanto, CV Fauna, CV Sauces, CV El Parque). Because of the randomization, the sample of veterinary centers chosen is heterogeneous in terms of the age of the veterinary team, medical equipment, knowledge, and geographical location.

All of them were provided with the available documentation about the biomaterial, and the handling protocol was explained to the staff. Material of different grain sizes (diameter range = 500–1,000 and 1,000–2,000 μm) was supplied to facilitate its use and adapt it to the size of the defect to be filled. They were also provided with a list of techniques in which the use of BV would be indicated.

At the end of each procedure, the veterinarians had to fill out a form (Supplementary Material 1) about the clinical use of the biomaterial (one per case). The results of this survey, along with the x-rays, were analyzed and compared by a committee of two outside blind experts who came to a consensus about whether the evolution was satisfactory or not.

### Clinical Cases and Criteria Selection

The legal owners of the animals signed an informed consent about the use of BV as a part of the treatment.

Twenty-four clinical cases (20 dogs and 4 cats) have been reported in which bone substitute of marine origin has been used to improve bone healing and fill critical defects. Fourteen of the cases were treatment of fractures, nine arthrodesis, and a benign bone cyst.

The patients were selected strictly according to their clinical status and a set of established criteria: patients of any age, sex, or weight; without any disease or systemic infection; and skeletally mature. The exclusion criteria were disease or systemic infection, malignant tumors, severe renal dysfunctions, a greater anesthetic risk, and animals with uncontrolled bone metabolism.

### Radiographic Evaluation

Radiographic images were taken before surgery to evaluate the injury and, shortly after, to evaluate the success of the procedure. To assess the evolution of the patients' progress, radiographic assessments were performed at 2, 4, 8, and 12 weeks after the surgery. Two radiographic views were made (anteroposterior and lateral). The follow-up consultations included a general physical examination, control radiographs, and feedback with the client to monitor the process. Each radiograph was evaluated by a stage score of 1 to 5 points to set the consolidation time (1: not visible callus formation; 2: barely visible callus formation; 3: scattered, not homogeneous callus; 4: uniform, mature callus formation; 5: very active, hyperthrophic callus formation) (29). The results were evaluated by the veterinary specialist of the entity and later by a panel of two blind outside experts.

### Epidemiological Survey

An epidemiological survey (Supplementary Material 1), as stated above, was sent to all the veterinarians for each case, including the patients' clinical history (anamnesis, diagnosis, surgical treatment, and progress), functional recovery and information related to the biomaterial and its handling (grain size used, difficulty in using it, mixing with another material or substance, advantages over other fillings), and a radiographic report. Finally, the veterinarians provided a final report on their experience in the clinical use. Later, the obtained information was reviewed by a panel of blind independent experts to see the suitability of the treatment and its correct evolution.

### Functional Recovery

Functional recovery was evaluated by the clinicians of the centers in each revision, using a simple scale (1–9, 11–16) with three levels: good (12–16), regular (6–9, 11), and poor (1–5), indicating total, partial, and no recovery of function, respectively. The criteria that were evaluated were as follows: lameless, pain on palpation, and weight-bearing (Table 1) (30, 31).

**Table 1:** Functionality recovery scoring system for assessing patients (30, 31).

**Criteria**	**Score**	**Clinical evaluation**
Lameless	1	Not walk
	2	Severe limp when walking
	3	Moderate limp when walking
	4	Slight limp when walking
	5	No limp. Walk normally
Pain on palpation	1	Patient cannot be palpated
	2	Severe signs; patient vocalizes or becomes aggressive
	3	Moderate signs; patient pulls limb away
	4	Mild signs; patient turns head in recognition
	5	None
Weight-bearing	1	Non–weight-bearing standing and walking
	2	Partial weight-bearing standing; non–weight-bearing walking
	3	Partial weight-bearing standing; non–weight-bearing walking
	4	Normal standing; favors affected limb when walking
	5	Equal on all limbs standing and walking

### Statistical Analysis

A statistical analysis was carried out with the consolidation time in search of statistically significant differences regarding sex (males and females), weight (three groups: (a) <5 kg, (b) 5–20 kg, (c) >20 kg), and procedure (internal or external fixation and arthrodesis).

Statistical analysis was performed with the computer program SigmaPlot® 12.5 for Windows (Systat Software Inc., San José, CA, United States). A value of *p* < 0.05 was considered statistically significant. The descriptive study of the population was shown as the mean ± SD. The normality of variances was assessed using the Shapiro–Wilk test. To determine the differences between the groups for non-categorical variables (weight), a one-way analysis of variance was done. Categorical variables (sex and procedure) were assessed using *t*-test and one-way analysis of variance.

## Results

Nineteen cases completed the study (18 dogs and 1 cat; 12 males and 7 females; 11 fractures, 7 arthrodesis, and 1 bone cyst) (Table 2). The remaining five were excluded because they did not complete the radiographic follow-up (three cats and two dogs; three arthrodesis and two fractures). The age of the canine patients ranged from 1 to 15 years (average age of 7 years), being 10 males and 8 females, with weight ranging from 2.5 to 36 kg (average of 16.4 kg). The feline patient was a 3-year-old male and of 3.5-kg weight. In all cases except for one (case 11), the functional recovery was good, and no complications were recorded.

**Table 2:** Summary of patients treated with BIOFAST-VET.

**No**	**Species/Breed Age/Gender/Weight**	**Orthopedic problem**	**Type of surgery**	**Grain size**	**Consolidation time (weeks)**	**Functional recovery and complications**
1	Dog/English Setter/10/F/30 kg	Old failure due to an arthrodesis infection with steppped plate in carpal joint	Carpus arthrodesis	1–2 mm	7	Good, none
2	Dog/German Shepherd/Adult/M/35 kg	2 fractured metatarsals	Tarsal arthrodesis	1–2 mm	8	Good, none
3	Dog/Yorkshire/Adult/M/2.5 kg	Distal third fracture of radius and ulna	ESF	1–2 mm	4	Good, none
4	Dog/Crossbreed/Adult/M/15 kg	Slightly comminuted diaphyseal fracture of radius and ulna	ISF	0.5–1 mm	4	Good, none
5	Dog/Yorkshire/Adult/M/2.5 kg	Distal third fracture of radius and ulna	ISF	0.5–1 mm	5	Good, none
6	Dog/Crossbreed 1/F/13 kg	Highly comminuted femoral diaphyseal fracture	ESF *Tie in*	1–2 mm	6	Good, none
7	Dog/Crossbreed Adult/F/8 kg	Transverse fracture of radius and ulna	ISF	1–2 mm	4	Good, none
8	Dog/Crossbreed Adult/F/11 kg	Radius and ulna fracture	ISF	0.5–1 mm	8	Good, none
9	Dog/Crossbreed Adult–F−12 kg	Intercondylar fracture	ISF with 2 condylar needles	1–2 mm	8	Good, none
10	Dog/Crossbreed Adult/M/12 kg	Tibia and fibula fracture	ISF	1–2 mm	7	Good, none
11	Dog/Crossbreed Adult/M/16 kg	Old femoral fracture by firearm	ISF	1–2 mm	—	Poor, infection and biomaterial expulsion
12	Dog/German Shepherd/8/M/36 kg	Highly comminuted open fracture of the tibia	Tarsal arthrodesis	1–2 mm	4	Good, none
13	Dog/Teckel 2–F/10 kg	Benign bone cyst	Bone biopsy and defect filling	0.5–1 mm	8	Good, none
14	Dog/Crossbreed/8/F/3 kg	Open tibial and tarsal fracture	Tarsal arthrodesis	1–2 mm	8	Good, none
15	Dog/Boxer 1.5/M/29 kg	Severe injuries to the tendons of the tarsal joint	Tarsal arthrodesis	1–2 mm	6	Good, none
16	Dog/Boxer 1.5/M/29 kg	Severe distal third injuries to the radius, carpus and metacarpus	Radial, carpal and metacarpal panarthrodesis with circular ESF	1–2 mm	6	Good, none
17	Cat/European Common/3/M/3.5 kg	Tibial fracture	ESF	1–2 mm	4	Good, none
18	Dog/English Setter/3/M/27 kg	Severe radiocarpal joint injuries	Carpal arthrodesis	1–2 mm	8	Good, none
19	Dog/Crossbreed/14/M/17 kg	Multifragmentary femoral fracture	Double ISF	1–2 mm	8	Good, none

The statistical analysis was carried out only in dogs, because the sample in cats was finally not representative (one case), and only with arthrodesis and fractures (with external and internal fixation). Given the hypothesis whether sex or weight influences the time of consolidation, no statistically significant differences were found. Also, no statistically significant differences were found when comparing the procedures made (Table 3).

**Table 3:** Statistical parameters about the time of consolidation compared with the sex, weight, and procedure (*p* < 0.05 was considered statistically significant).

	**Variable**		**Mean and SD (weeks)**	***p***	
Sex	Males		6 ± 1.7	0.233	
	Females		7 ± 1.52		
Weight	Group (a): <5 kg		5.67 ± 2.08		
	Group (b): 5–20 kg		6.63 ± 1.77	0.714	
	Group (c): >20 kg		6.50 ± 1.52		
Procedure	Fractures	Internal fixation	6.14 ± 1.77	0.436	
		External fixation	5.00 ± 1.41		0.433
	Arthrodesis		6.71 ± 1.50	—	

**Figure 1:**
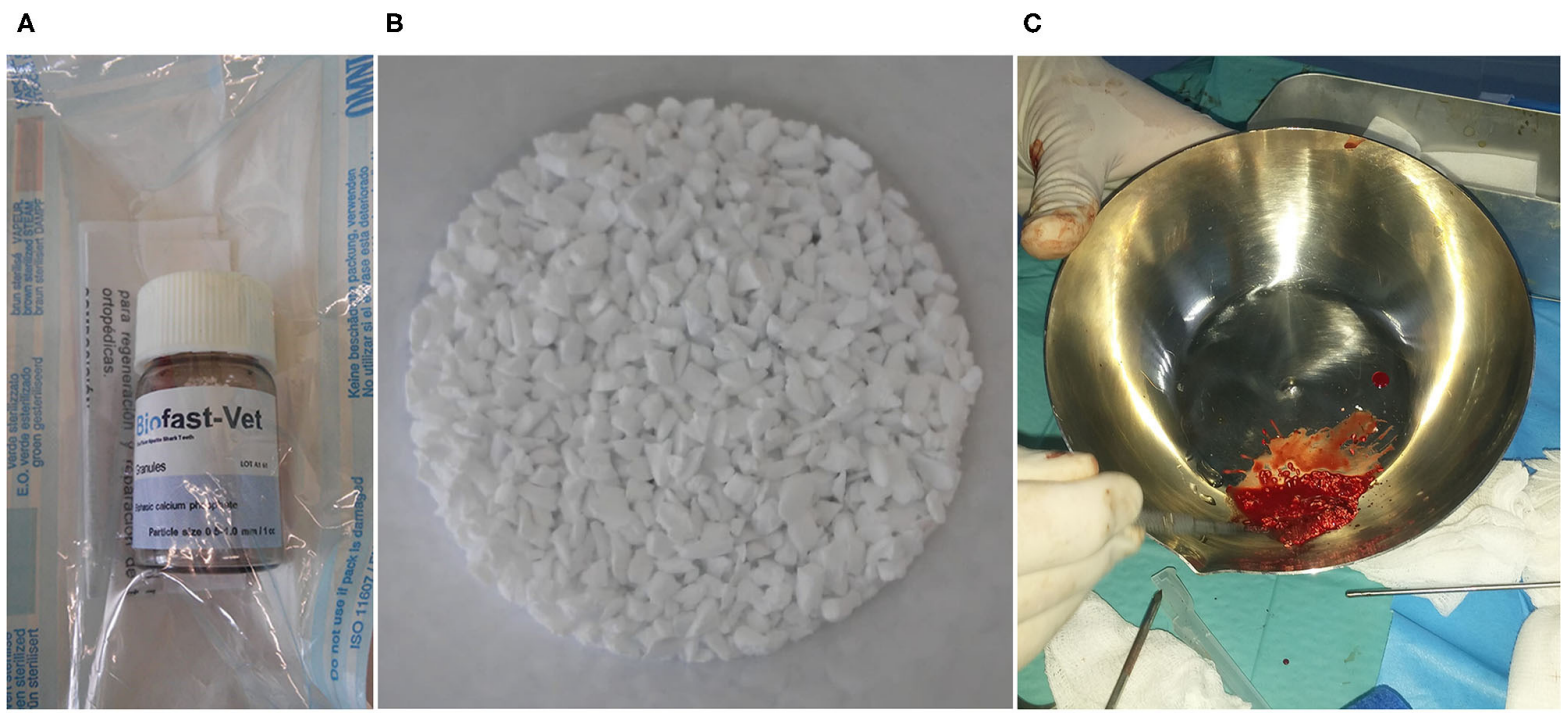
Biomaterial vial **(A)**, biomaterial granules **(B)**, preparation of the biomaterial mixture for intraoperative application with autologous blood **(C)**.

The bone substitute of marine origin (Figure 1) was applied mixed with the patient's own blood, previously extracted. In all cases, BV was used to improve bone healing, fill in critical defects, and complement the recommended procedures.

The suitability of the bone substitute of marine origin was studied in different types of orthopedic pathologies in veterinary field. In specific, BV was used as a bone substitute and to perfect traditional methods (bone defects caused by cysts, external and internal fixation of breakages, and arthrodesis).

In all of the cases, there were no reports on adverse reactions at the grafting site or at systemic level related to biomaterial. Except for case 11, there were no records of postoperative infection or foreign body reaction, regardless of the amount of grafted material. In case 11, because it was an old fracture by firearm and remitted from a less specialized clinic, the infection was not treated properly from the beginning, and the filling was not successful, producing a fistula after 2 weeks. The group that reviewed the case considered that, in this case, the use of the biomaterial had been contraindicated.

The efficacy of surgical treatments was performed by clinical examination and radiographic evaluation at the times established. In most of the successful cases, postsurgical x-ray controls indicated high rates of bone regeneration, being the mean consolidation time 5.94 weeks (ranging from 4 to 9 weeks). Except in case 11, all the bone defects healed without complications 12 weeks after the procedure.

The retrospective study of the questionnaire revealed that all the veterinarians who used BV agreed that it was an easy to use biomaterial. They asserted that the consolidation time was reduced, as well as the acceleration of joint fusions in cases of arthrodesis.

### Clinical Cases Description

**Figure 2:**
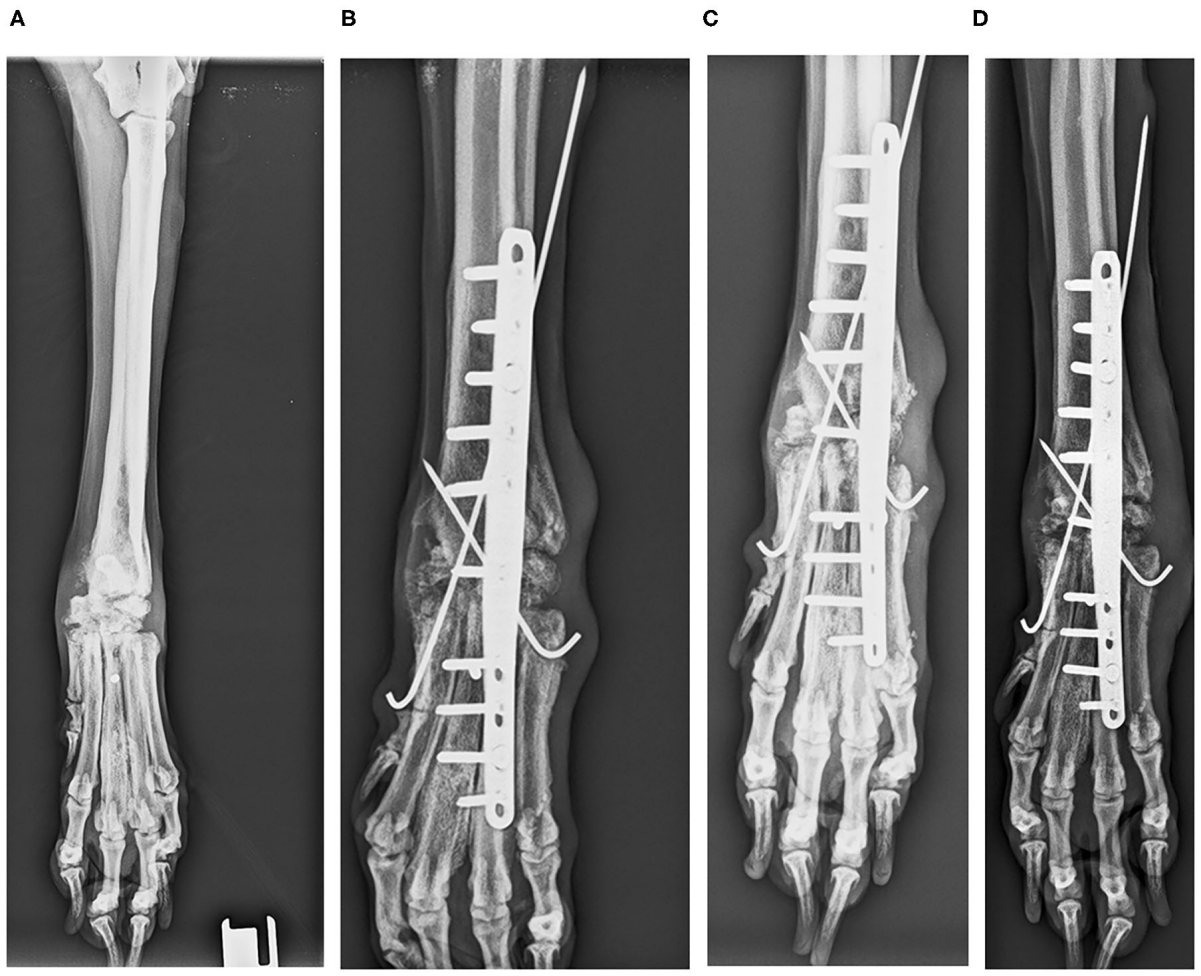
Case 1. Dorsopalmar radiographic images of the carpal joint infection. Preoperative radiographic image **(A)**; postoperative radiographic image **(B)**; Postoperative control 4 weeks after surgical procedure **(C)**; postoperative control 12 weeks after surgical procedure **(D)**.

**Clinical Case 1**. Dog, English setter breed, female of 30 kg, presented by an old failure due to a panarthrodesis infection with stepped plate in the carpal joint. It was decided to place a titanium plate on the dorsal side, using two crossed needles and a BV graft. The results were good, and the bone substitute helped the bone to consolidate faster (Figure 2).

**Clinical Case 2**. Dog, German shepherd breed, adult male of 35 kg, presented an old fracture (3 months) of two metatarsals. We performed tarsometatarsal arthrodesis from the calcaneus to the metatarsals by a lateral approach and using a BV graft. The radiographic evaluation and the clinical examination were performed up to 8 weeks, a good and effective osseointegration being observed.

**Clinical Case 3**. Dog, Yorkshire breed, adult, male of 2.5 kg, presented a distal third fracture of the radius and ulna. An external type II fixator with five metallic thread needles and a BV graft is placed in the fracture site. It is a case in which the external fixation is not ideal, since the literature (32–35) describes the possibility of a non-union in fractures of the third distal of the radius in small breeds, being able to cause deformations, and requiring new procedures and even amputations. The use of the bone graft of marine origin resulted in rapid ossification of the fracture, within 4 weeks after the surgery, a complete ossification being observed at the fracture site at 6 weeks.

**Figure 3:**
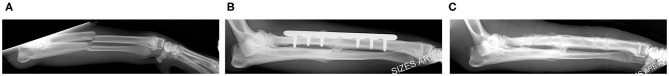
Case 4. Mediolateral and laterolateral radiographic images of radius and ulna fracture. Preoperative radiographic image **(A)**; postoperative control 4 weeks. Consolidation is observed **(B)**; postoperative control 8 weeks. Remodeling is observed **(C)**.

**Clinical Case 4**. Mixed-breed dog, adult, 15-kg male, presented a slightly comminuted transverse diaphyseal fracture of the ulnar and radius of the left limb. It was stabilized with an internal fixation plate and a BV graft at the fracture site. At 4 weeks, consolidation was observed, and at 6 weeks, the plate was removed. At 8 weeks, the fracture site was completely remodeled (Figure 3).

**Clinical Case 5**. Dog, Yorkshire breed, adult, male of 2.5 kg, presented non-union in a distal third fracture of the radius and ulna, a relatively large isolated bone fragment being observed. An internal fixation plate was placed, the necrosed bone fragment is removed, and BV graft was added to keep the length of the bone. The bone substitute provided a faster consolidation of the fracture. Eight weeks later, radiographs revealed a complete ossification of the fracture site.

**Clinical Case 6**. Mixed-breed dog, female of 8 kg and 1 year old, presented a highly comminuted femoral diaphyseal fracture in the left posterior limb. It was a complex fracture in a growing dog, which occurred 5 days before the procedure. It was decided to perform a functional reduction and stabilized the fracture with an external tie-in fixator and a BV graft. Two weeks later, an early ossification was radiographically observed, integrating the graft grains perfectly into the new tissue.

**Figure 4:**
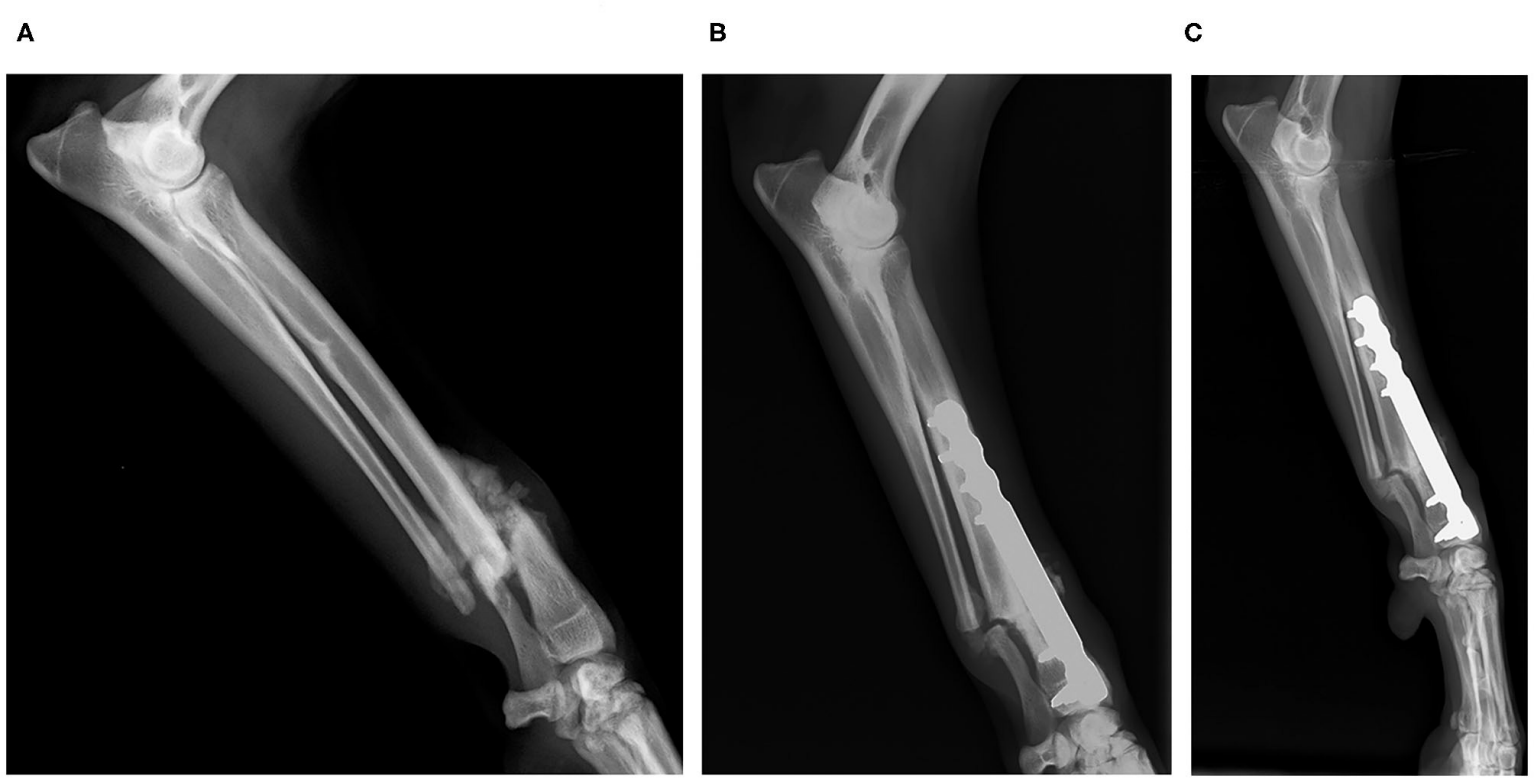
Case 7. Mediolateral radiographic images of the radius and ulna fracture. Preoperative radiographic image **(A)**; postoperative control 4 weeks **(B)**; postoperative control 8 weeks. Remarkable ossification is observed. The material has been fully integrated and reabsorbed. The ulna is not ossifying because it was not aligned properly **(C)**.

**Clinical Case 7**. Mixed-breed dog, adult, female of 8 kg, presented malunion of a transverse fracture of radius and ulna in the left anterior limb. Non-functional bone tissue formation was observed. The fragments were separated with a surgical saw and aligned correctly. Finally, an internal fixation plate and BV graft were placed to favor the ossification process. The patient evolved favorably. At 7 weeks, a remarkable advance in the radial ossification process was observed radiographically. The filling was practically reabsorbed and integrated into the new tissue. The ulna did not ossify because it was not correctly aligned (Figure 4).

**Clinical Case 8**. Mixed-breed dog, adult, female of 11 kg, presented a radius and ulna fracture. After studying the situation, it was decided to use an internal fixation with a locking plate and a BV graft in the fracture center. Eight weeks later, radiographs showed a complete ossification of the fracture site.

**Clinical Case 9**. Mixed-breed dog, adult, female of 12 kg, presented an intercondylar humerus fracture of the left forelimb. The fracture was stabilized by placing two needles and an internal fixation plate with the BV graft. Radiographs revealed a correct evolution and consolidation at 7 weeks.

**Clinical Case 10**. Mixed-breed dog, adult, male of 12 kg, presented a fracture of the tibia and fibula. It proceeded to stabilize the tibia fracture using an internal fixation plate with six screws. In this case, no follow-up radiographs are available, but the owners stated that the animal had been using the paw for support and did not show any pain 2 weeks after the procedure.

**Figure 5:**
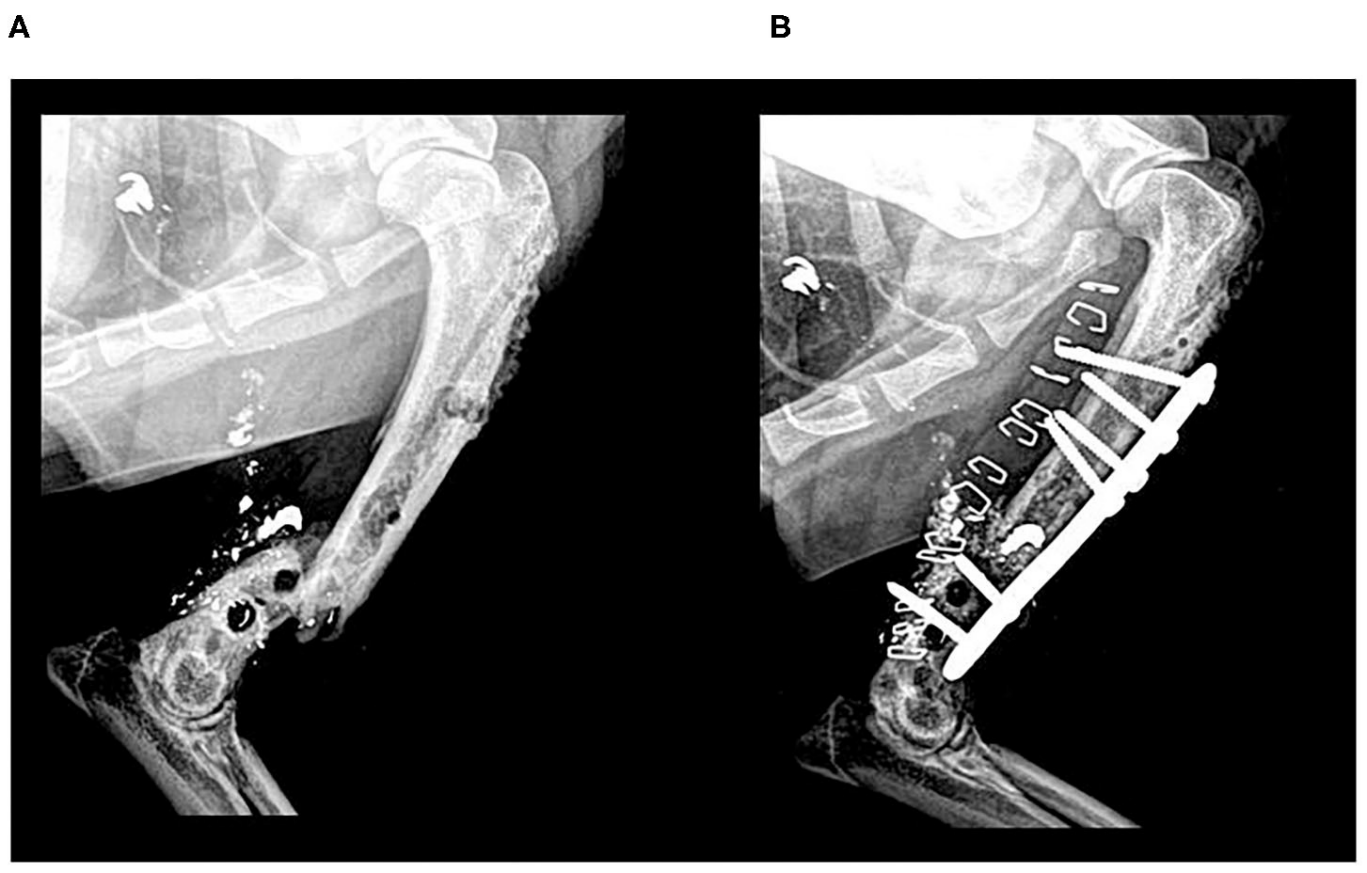
Case 11. Mediolateral radiographic images of an old femoral fracture by firearm. Preoperative reintervention radiographic image **(A)**; postoperative control 2 weeks. Suppuration and elimination of the biomaterial can be observed **(B)**.

**Clinical Case 11**. Mixed-breed dog, adult, male of 16 kg, presented an old humerus fracture by firearm. Fractures caused by firearm represent a delicate situation in traumatology (36). In general, they are contaminated, so they tend to become infected, being complicated to treat. In this case, the fracture was old, and the patient was referred from a poorly specialized clinic where an attempt has been previously made to stabilize the fracture with an external fixator. The fracture was stabilized with an internal fixation plate and a BV graft aimed at promoting ossification. Unfortunately, after 2 weeks, the infection continued, and the bone substitute was expelled from the body with the suppuration. In this case, the use of BV did not have any result because the local infection had not been properly treated well (Figure 5).

**Figure 6:**
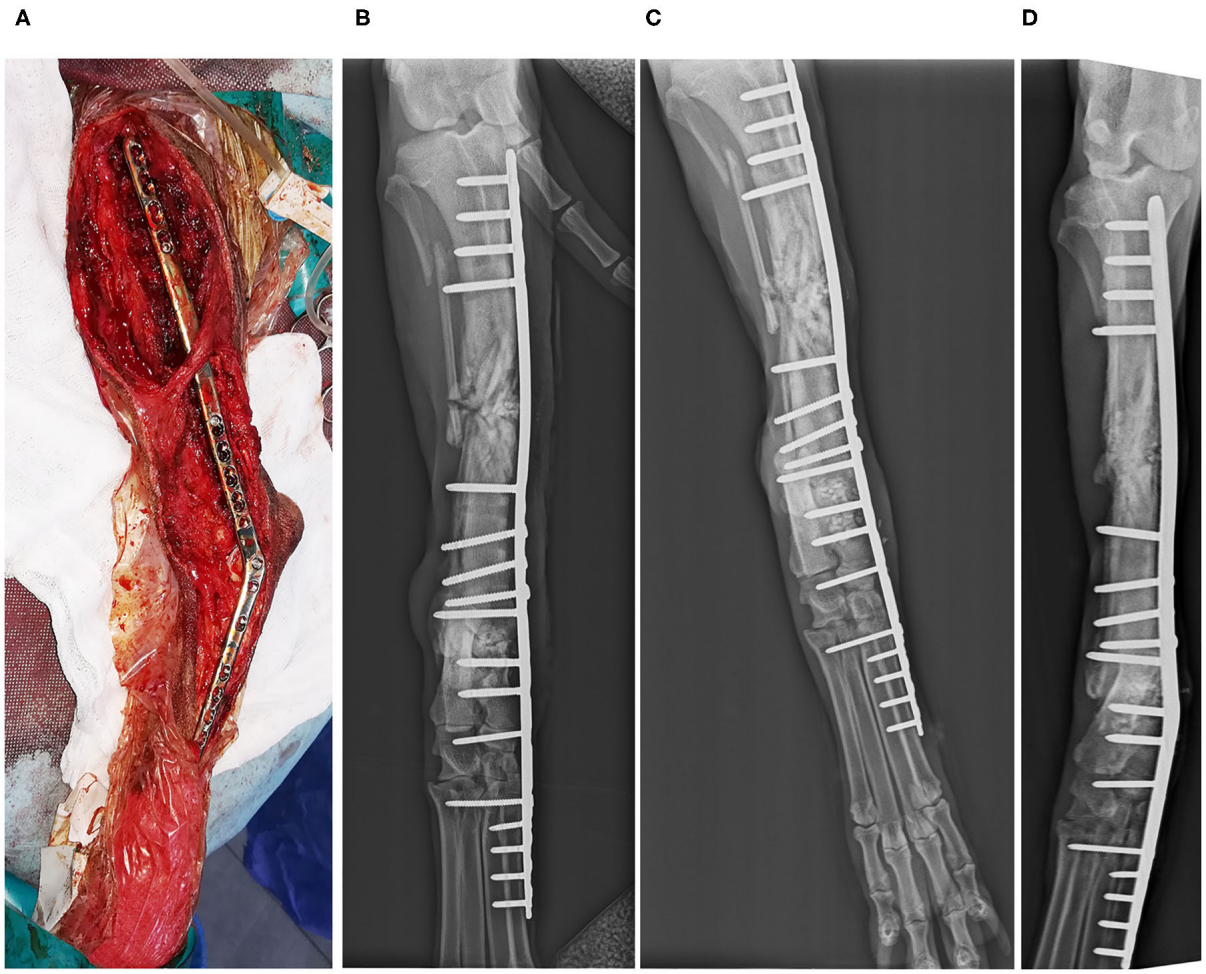
Case 12. Highly comminute open fracture of the tibia correct by tarsal arthrodesis. Intervention **(A)**; postoperative image **(B)**; postoperative control 4 weeks **(C)**; postoperative control 8 weeks, perfect ossification is observed **(D)**.

**Clinical Case 12**. Dog, German shepherd breed, male of 36 kg, presented a highly comminuted open fracture of the tibia, with great destruction of soft tissue. In the first procedure, a wall locking plate was placed. A week after that, the patient presented an angular deviation of the limb and plantigradism, because the plate was bent because of the empty holes, and, in addition, presented a lesion of the Achilles tendon, with rupture of the deep flexor. Subsequently, it was proposed to solve everything with surgery. A custom hybrid plate was designed, with locking screws, to solve the angulation and achieve a tarsal, since it was not possible to suture the tendon. The callus formed was broken to facilitate the alignment, and the BV graft was incorporated. The result was very good, an almost perfect alignment was achieved, and an almost perfect functionality was granted. Radiographs showed a complete ossification at 8 weeks (Figure 6).

**Figure 7:**
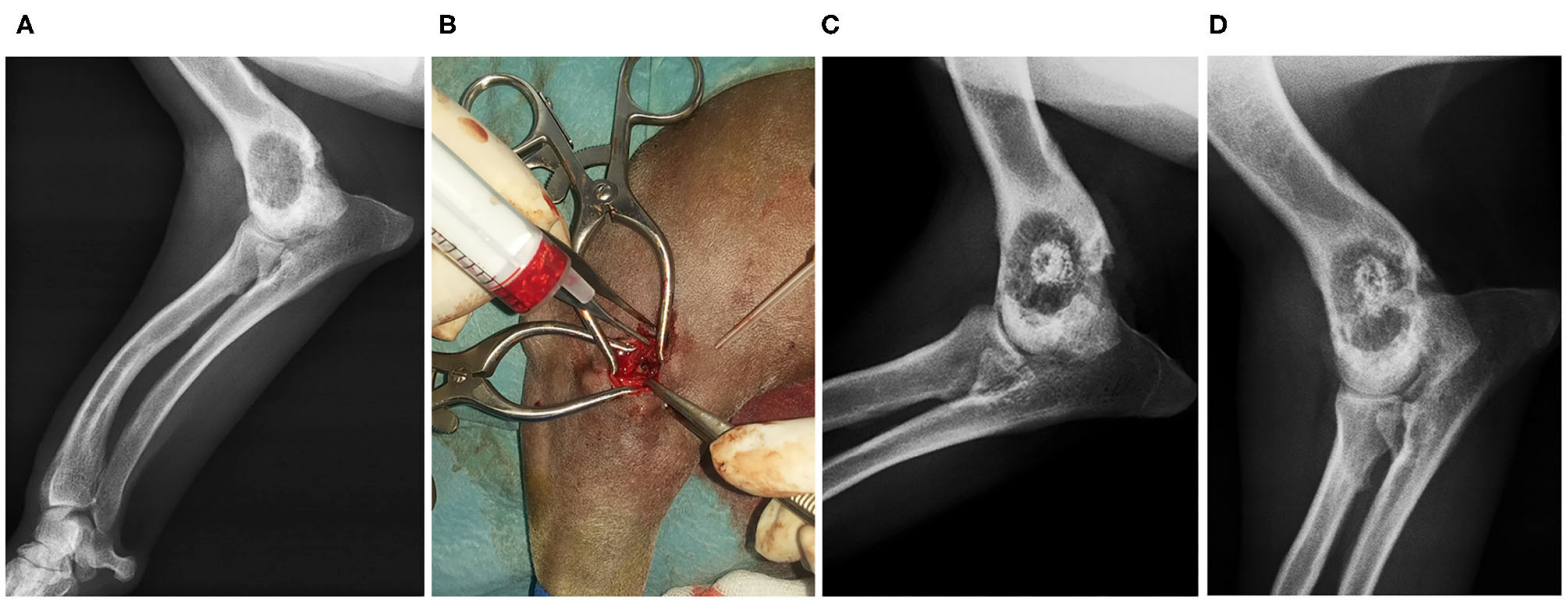
Case 13. Preoperative radiographic image of humerus benign bone cyst **(A)**; infiltration of the biomaterial **(B)**; postoperative control 4 weeks image **(C)**; postoperative control 8 weeks image **(D)**.

**Clinical Case 13**. Dog, Teckel breed, female of 10 kg, presented an expansive lesion in the distal metaphysis of the right humerus, with non-specific right axillary lymphadenopathy. Bone biopsy was performed, and a benign unicameral bone cyst was diagnosed. The defect was filled with a BV graft. Eight weeks later, radiographs showed good consolidation (Figure 7).

**Figure 8:**
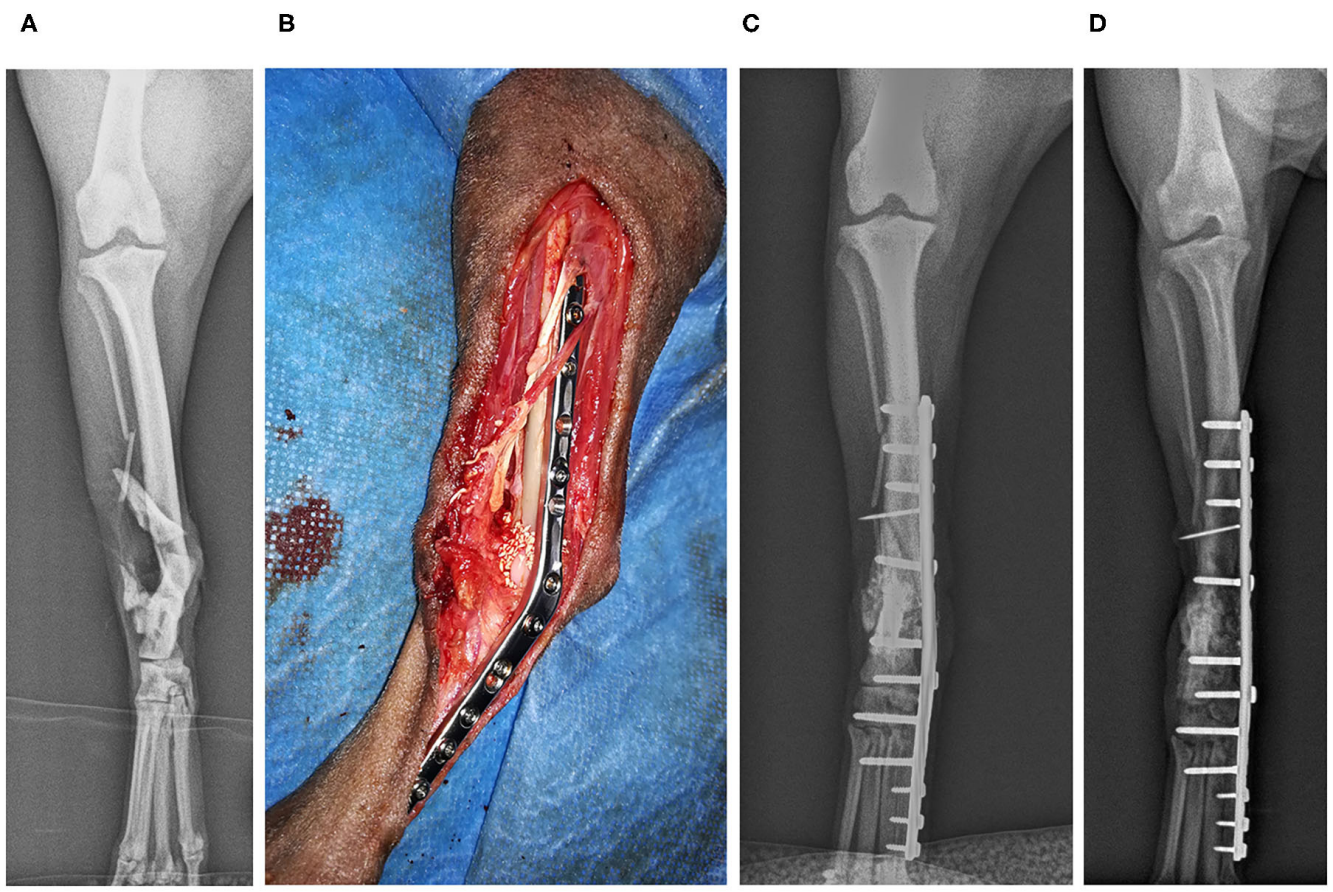
Case 14. Preoperative caudocranial radiographic image of an open tibial and tarsal fracture **(A)**; biomaterial graft intervention **(B)**; postoperative control 4 weeks. Good remodeling can be observed **(C)**; postoperative control 8 weeks. Completed remodeling can be observed **(D)**.

**Clinical Case 14**. Mixed-breed dog, female of 3 kg, was hit by a car and could not stand its posterior limbs. Radiographs revealed a distal fracture of the right tibia, with several fragments that reach the tarsal joint, destabilizing the joint. In the left posterior limb, the patient presented hip dislocation. It was decided to perform arthrodesis of the joint with an internal fixation plate and a bone graft. Four weeks later, radiographs revealed good remodeling, which was complete 8 weeks after the procedure (Figure 8).

**Clinical Cases 15 and 16**. Dog, Boxer breed, female of 29 kg, presented lesions in the left posterior limb, in the joint of the tarsus in the right posterior limb, and in the carpal joint in the right anterior limb.

- The left posterior limb had to be amputated because of irreversible injuries. An endoprosthesis and exoprosthesis was placed instead.
- In the right posterior limb, a partial arthrodesis of the joint was performed because of damage to the tendons. A BV graft was also placed.
- In the right anterior limb, there was no soft tissue coverage in the distal two-thirds of the radius. There was neither carpus nor metacarpals. An external fixator was placed; bandages and treatment were applied until the entire limb was covered with granulation tissue. At that point, a panarthrodesis was performed, using a circular fixator and a BV graft.

The use of BV provided very good results to accelerate the joint fusion because, in this case, there were three injured joints, and it was essential to achieve a quick consolidation to recover mobility as soon as possible. In addition, because of the serious soft tissue injuries, the implant of the right posterior limb was exposed, so it was vital to accelerate the consolidation to be able to remove the implant and thus allow the tissue recovery. In this sense, the use of BV was successful, allowing bearing weight normally on the affected limbs.

**Figure 9:**
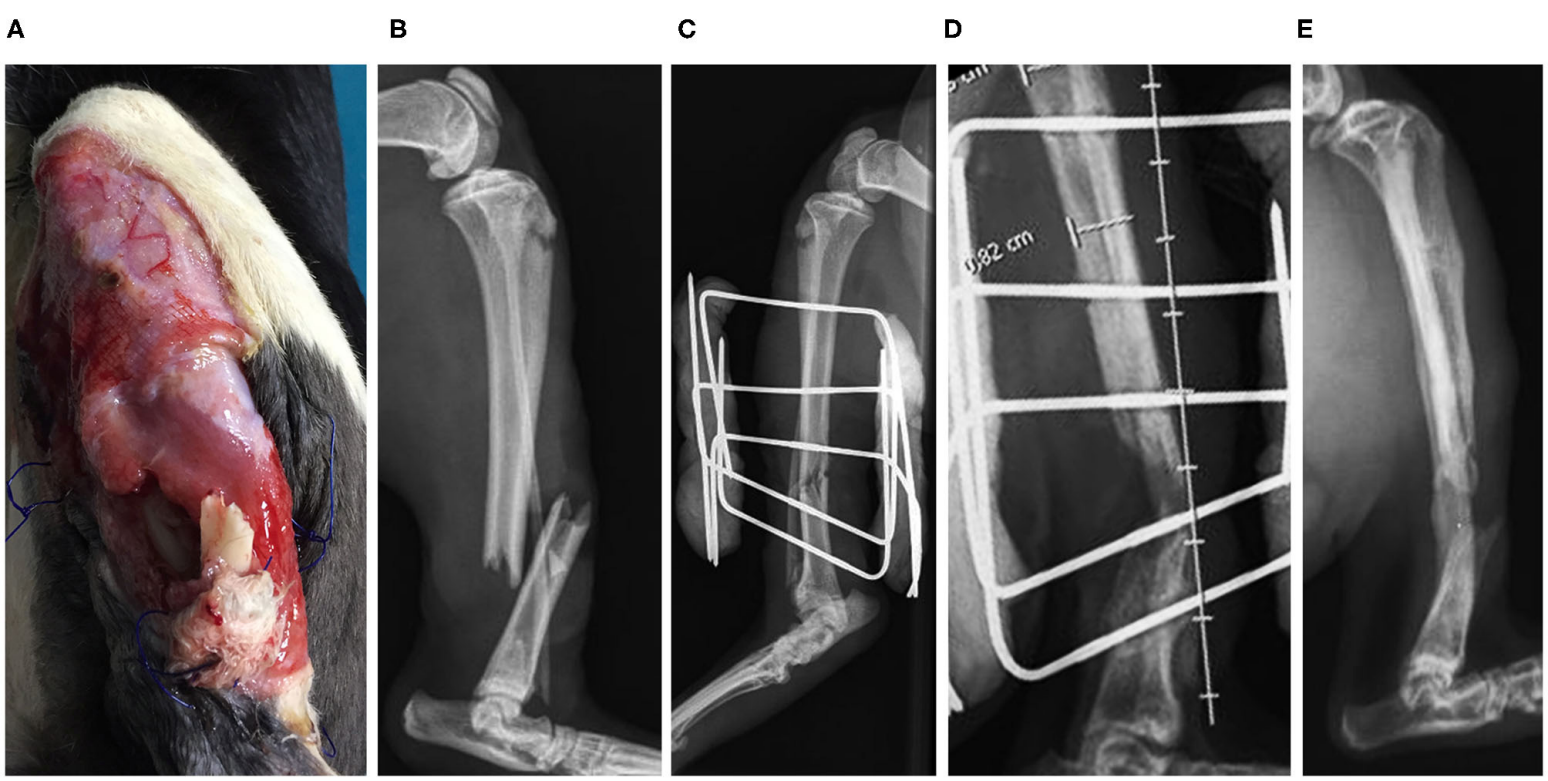
Case 17. Open tibial and ulna fracture in a cat **(A)**; preoperative radiographic image **(B)**; postoperative radiographic image **(C)**; postoperative control 4 weeks **(D)**; postoperative control 12 weeks **(E)**.

**Clinical Case 17**. Cat, European Common breed, male of 3.5 kg, presented a fracture of the tibia. We performed a reduction thereof, with an external fixator and a BV graft. At 8 weeks, a correct remodeling was observed radiographically, but the external fixator was not removed until after 12 weeks (Figure 9). In this case, fracture has healed with axial distortion with help of a BV.

**Clinical Case 18**. Dog, English setter breed, male of 27 kg, presented severe carpal joint lesions. During the first procedure, it was decided to place an internal fixation plate carrying out a carpal joint arthrodes, but 1 week after the procedure, the implants had to be removed because of infection. Three weeks later, the second procedure was performed, in which another plate was placed on the dorsal side, employing a BV graft, favoring a quick ossification. This allowed the removal of the implants earlier than usual (after 2½ months) and the beginning of rehabilitation.

**Figure 10:**
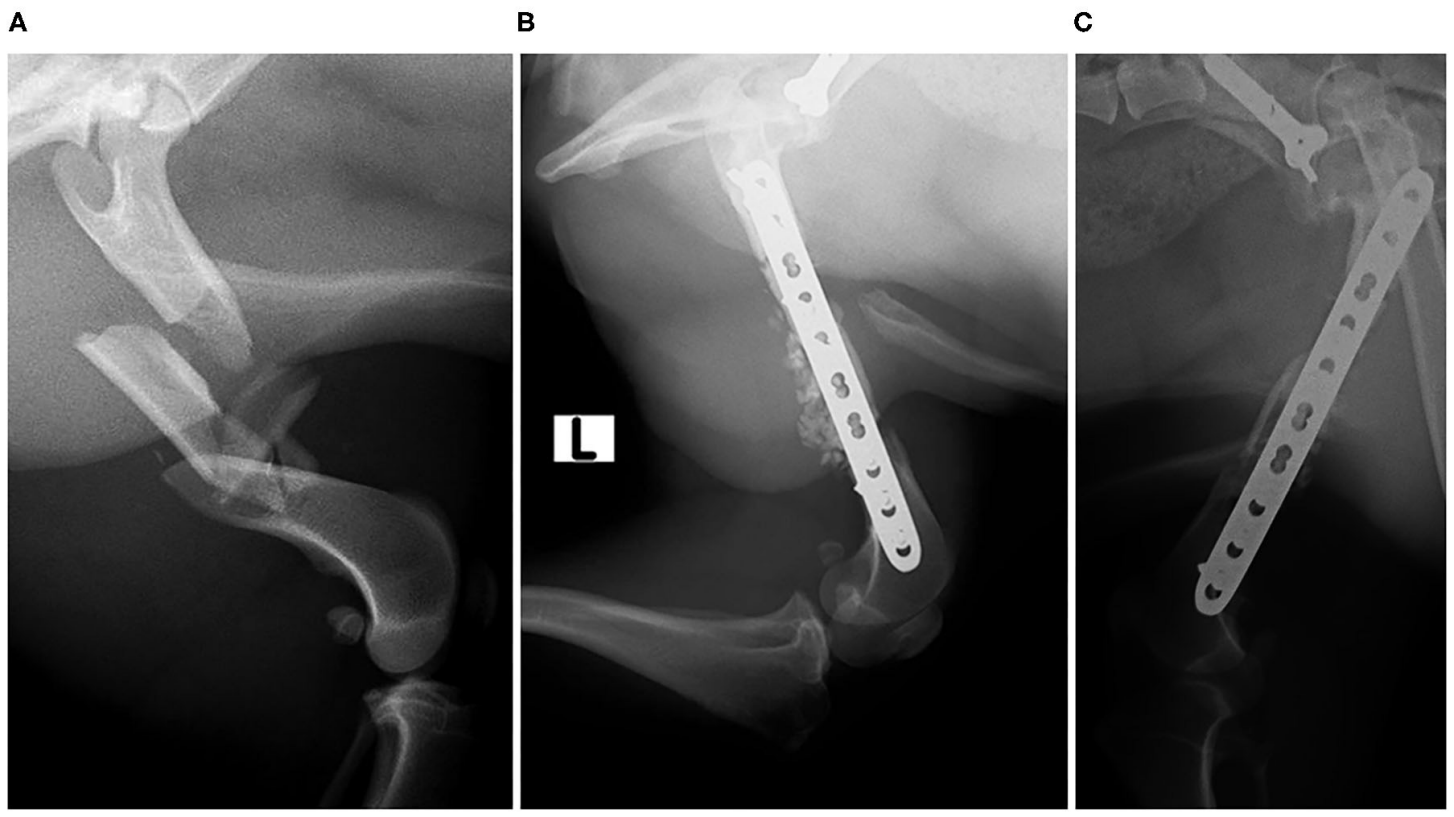
Case 19 Preoperative radiographic image of a multifragmentary femoral fracture **(A)**; postoperative control 4 weeks **(B)**; postoperative control 12 weeks **(C)**.

**Clinical Case 19**. Mixed-breed dog, male of 17 kg, presented with a multifragmentary fracture in the left femur. It was a very unstable fracture that applied load on the osteosynthesis implant in an extreme way, reducing its useful life. Therefore, a BV graft was added to promote rapid ossification and to reduce the load on the implant. BV met the expectations, and at 4 weeks, the patient showed a good evolution and perfect support, despite a very serious initial prognosis (Figure 10).

## Discussion

The present work demonstrated that porous globular bioapatites are viable for use in veterinary orthopedics. In all cases, there were no reports on adverse reactions at the grafting site or at systemic level. Except for case 11, there were no records of postoperative infection or foreign body reaction, regardless of the amount of grafted material. In case 11, because it was an old fracture by firearm and remitted from a less specialized clinic, the infection was not treated properly from the beginning, and the filling was not successful, producing a fistula after 2 weeks. Fractures caused by firearm represent a delicate situation in traumatology (36). The group that reviewed the case considered that, in this case, the use of the biomaterial had been contraindicated.

Fractures are the most common large-organ traumatic injuries (37). The normal and physiological reaction when a fracture occurs includes a series of events: initial inflammatory reaction, establishment of soft and subsequently hard callus and, finally, bone remodeling (38, 39). In cases where this physiological process does not occur, the surgery is indicated (40). Insufficient blood nutrition, a disease that systematically affects, or an infection of the bone callus can have a negative effect on the regeneration of the bone, which can trigger a non-union (41). In those situations where healing is not achieved, the use of bone substitutes is indicated (42). The autologous graft remains the gold standard in these types of situations, because it does not trigger immunological reactions, provides growth factors to help osteoinduction and is also part of the structure of the new bone (40, 43, 44). Nevertheless, the use of autologous bone has disadvantages: longer anesthesia, the availability of autologous bone is limited, high morbidity of the donor site (risk of intraoperative bleeding, pain, possibility of stress fractures), increased chances of local infection, and risk of failure (5, 45–47). It should also be noted that the amount of autologous bone collected and the viability of the cells collected are limited, which limits the application in situations where defects are critical (44). These disadvantages can be avoided by using synthetic bone substitutes instead of autologous bone. One of the advantages of these bone substitutes is their ease of storage (43, 48, 49). In this report, we presented a series of cases in which the BV bone mineral substitute was shown to be an effective alternative and without adverse effects. This biomaterial is an excellent ceramic scaffold to promote and strengthen the process of bone regeneration. BV contains a large amount of HA, which is one of the main components of the bone mineral matrix (50).

One of the challenges of using synthetic bone substitutes is maintenance within the defect. The mixing of BV with the patient's own blood achieved the desired effect, and the escape of the biomaterial was kept in the correct place. In addition, cell congregations within blood clots are bioactive potentials that favor the cure of the defect, promoting improved remodeling (51). The mixing of the biomaterial with platelet-rich plasma could also have been interesting, given the good results obtained in a study in which it was used for the treatment of fractures in dogs (29).

Other calcium phosphate ceramics have previously been used as bone substitutes in the veterinary field, obtaining good results in terms of improving the quality of life of the patient, speed of bone healing, and decrease in morbidity. In the mentioned research study (4, 48), the effectiveness of the material in appendicular and maxillary/mandibular bone defects was studied. The patients did not present any adverse effects at the local or systemic level, and the functional recovery was good in all cases, as in the present study. Despite being a preliminary study, in the present study, shorter consolidation times were obtained (5.94 vs. 9.07 weeks). However, controlled studies would be needed to give more weight to this premise.

Despite not having found statistically significant differences in the comparison of the consolidation time with sex, weight, and procedure, a slight trend can be observed in a shorter consolidation time in dogs of <5 kg and in males.

The present study has some limitations. The diagnostic use of computed tomography would have been interesting to assess the density of postsurgical bone mineralization and at 12 weeks in the defect. The use of questionnaires in owners could have provided more quantitative data regarding the recovery of the animal. No controlled group was available. The statistical analysis of the age variable could have provided more quantitative data, but it could not be done because all ages were not available.

Seeing the promising results have been obtained in this preliminary study, it would be very useful to carry out controlled clinical trial to complement and provide more quantitative and comparative data about the study of this biomaterial.

## Conclusions

In the present study, the viability of using globular porous bioapatites of marine origin in the veterinary field has been demonstrated. This biomaterial is presented as a very suitable candidate for orthopedic surgery in the veterinary field. It has been used successfully in 18 cases in which no local or systemic adverse reactions related to the biomaterial have been detected. Regarding the consolidation time, it is observed that the use of this biomaterial in dogs with arthrodesis and fractures can reduce it compared to other biomaterials.

However, controlled clinical studies are needed to evaluate the behavior of the biomaterial in different clinical settings.

## Data Availability Statement

The relevant data for the investigation are collected in the text, tables and figures. The rest of the data are personal information of the patients' owners, being sensitive, and therefore, confidential.

## Ethics Statement

The animal study was reviewed and approved by Xunta de Galicia. Written informed consent was obtained from the owners for the participation of their animals in this study.

## Consent for publication

All authors of this research consented to the publication of this paper.

## Author Contributions

PG-F, JS, and RO obtain and study the biomaterial and their properties (morphology, characterization, and composition) and choose the clinics where it was to be studied clinically. MG-G, FM, and AG-C recompile all information about the cases and analyze the suitability of the treatment and its correct evolution. MG-G carried out the manuscript design and drafted it. All authors have read and approved the final manuscript.

## Conflict of Interest

The authors declare that the research was conducted in the absence of any commercial or financial relationships that could be construed as a potential conflict of interest.

## Abbreviations

μm: micrometer
°C: degrees Celsius
BMP: bone morphogenetic proteins
BV: BIOFAST-VET
h: hour
HA: hydroxyapatite
Hz: Hertz
kg: kilogram
mm: millimeter
min: minute
P: significance level
TCP: tricalcium phosphate.
