# Supplementary material for: Application of Shark Teeth–Derived Bioapatites as a Bone Substitute in Veterinary Orthopedics. Preliminary Clinical Trial in Dogs and Cats
Source: Front Vet Sci. 2020 Oct 28;7:574017. doi: 10.3389/fvets.2020.574017 (PMC7655648; doi:10.3389/fvets.2020.574017)
Supplement: Supplementary file 1 [file Data_Sheet_1.DOCX]

| **Case n:**  **Supplementary material 1**. Epidemiological survey and case data collect |  | | | | |
| --- | --- | --- | --- | --- | --- |
| **Specie :** | **Breed:** | **Activity level:** | | **Age:** | |
| **Clinical History** |  | | | | |
| **Description of the intervention** |  | | | | BIOFAST-VET  Grain size used:  0.5- 1mm ☐  1mm – 2mm ☐  Quantity:  < 1cc ☐  1cc ☐  2cc ☐  > 2 cc ☐ |
|  | | | | | |
| **Results and evolution** |  | | | | |
|  | | | | | |
| **Use of BIOFAST** | Handdling | | Dificulty of use Grade:  1 2 3 4  YES ☐ NO ☐  Mix with other material Which one:    YES ☐ NO ☐ | | |
|  | Advantages in relation to other fillers | | In this pathology it is always necessary to use a bone graft?  YES ☐ NO ☐  Compared with other bone grafts used in this pathology  Does the use of BIOFAST bring advantages?  YES ☐ NO ☐  What are the advantages of using BIOFAST compared to other bone fillers ?:  Reduces the ossification time of the fracture YES ☐ NO ☐  Improves the quality of bone repair YES ☐ NO ☐  Other comments: | | |
| **Veterinarian testimony and conclusions** |  | | | | |

**FUNCTIONALITY SCALE**

| **CRITERIA** | **SCORE** | **CLINICAL EVALUATION** |
| --- | --- | --- |
| Lameless | **1**  **2**  **3**  **4**  **5** | Not walk  Severe limp when walking  Moderate Limp when walking  Slight limp when walking  No limp. walk normally |
| Pain on palpation | **1**  **2**  **3**  **4**  **5** | Patient cannot be palpated  Severe signs; dog vocalizeas or becomes aggressive  Moderate signs; dog pulls limb away  Mild signs; dog turns head in recognition  None |
| Weight-bearing | **1**  **2**  **3**  **4**  **5** | Non-weight-bearing standing and walking  Partial weight-bearing standing; non-weight-bearing walking  Partial weight-bearing standing; non-weight-bearing walking  Normal standing; favors affected limb when walking  Equal on all limbs standing and walking |

**RADIOGRAPHIC ASSESSMENT**
